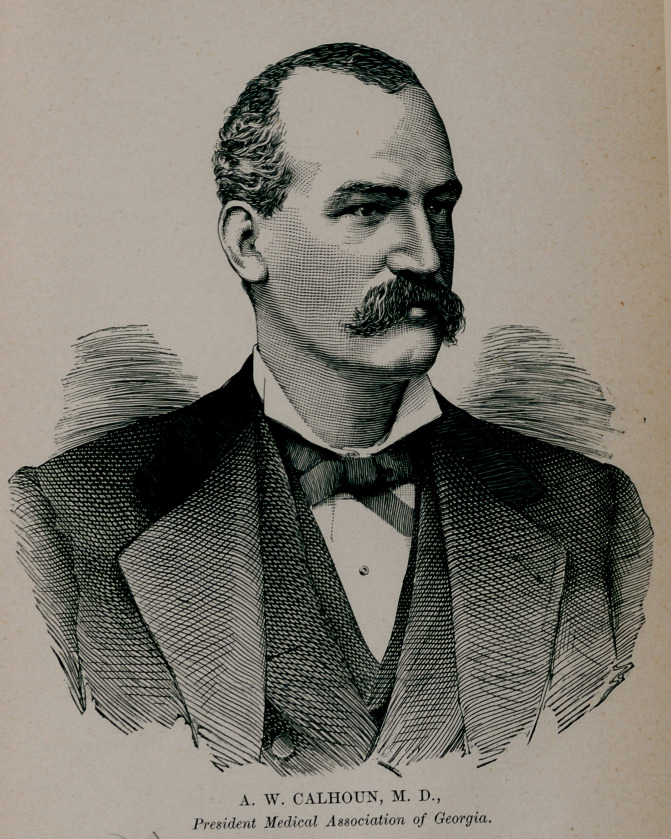# Hydrochlorate of Cocaine—A Clinical Lecture

**Published:** 1885-01

**Authors:** A. W. Calhoun

**Affiliations:** Professor Diseases of the Eye, Ear and Throat, Atlanta Medical College, Atlanta, Ga.


### HYDROCHLORATE OF COCAINE.

### ADDITIONAL CLINICAL OBSERVATIONS UPON THE NEW ANÆSTHETIC.

### A CLINICAL LECTURE.

---

BY A. W. CALHOUN, M. D.,

Professor Diseases of the Eye, Ear and Throat, Atlanta Medical College, Atlanta, Ga.

---

Reported for the ATLANTA MEDICAL AND SURGICAL JOURNAL.

The daily use of muriate of cocaine during the last month strongly confirms my views as to its efficacy as a local anæsthetic, as given in the report of the ten cases published in THE ATLANTA MEDICAL AND SURGICAL JOURNAL for December. That it deteriorates in time there can scarcely be any doubt. In using the first supply of the drug received, I found each day, while it lasted, that it took more time and more of the medicine to get the patient under its influence. I am inclined to the opinion that the addition of the purest boracic acid (10 grains to the oz.) would check this deterioration, and keep the solution as originally prepared. On account of the expense, the disposition to lose its strength, and the small amount required in each case, it would be well to have only a small quantity prepared at a time.

It has in every instance enlarged the pupil, but it affects the accommodation very slightly, if at all. For this reason alone it will become an important and useful medicine in eye affections, when it gets to be plentiful and cheap. The dilatation of the pupil is of short duration, lasting from three to six or eight hours. The conjunctiva and sclera become blanched, even the larger blood

A. W. CALHOUN, M. D.,

*President Medical Association of Georgia.*

vessels diminishing in size, clearly suggesting some of the happy results to be looked for when used upon inflamed surfaces.

Its anæsthetic effect is short-lived, continuing but gradually lessening for thirty or forty minutes, at the end of which time the sensibility of the eye is just as it was previous to the instillation of the fluid. Complete anæsthesia is attained at the end of ten to twelve minutes, by instilling two or three drops of the 4% solution into the eye every two or three minutes.

It temporarily, but effectually relieves pain; it completely controls photophobia, and enables the sufferer to open the eye and face to almost any light; it rids the patient of the fear of being hurt, and transforms him into a valuable assistant, and prepares the eye, even of children, to painlessly undergo operations which formerly necessitated the use of chloroform or ether. Besides, unlike most anæsthetics, it leaves behind no ill effects. Locally used, it is absolutely harmless.

Since the last report five cataract cases have been operated upon, two of which were children. The adults suffered almost no pain, and were themselves delighted with its success as an anæsthetic. The children submitted to the operations without hesitation, being assured it would not hurt them, simply giving evidence of fright during the progress of the operation. In this class of cases the cocaine will prove invaluable, as it enables the patient to remain absolutely quiet, and does away with the dangerous after-effects of chloroform or ether.

In *four cases of strabismus* there was no complaint of pain at all in cutting through the conjunctiva, but in severing the tendons of two of them there was complaint of great discomfort. In one of the two the advancement of the muscle was done, a tedious and trying procedure, and without cocaine, would have been exceedingly painful. Although the operation for strabismus is quickly made, I have my assistant to instill one drop into the eye every half minute or so, during the progress of the operation, with the decided effect of keeping the eye fully influenced.

A number of iridectomies (one on a child only 12 years old), and operations for closure of the tear canal, pterygium, and foreign bodies in the cornea have been made, and without exception, the eyes were cocainized and the operations completed with little or no pain.

The simple removal of foreign bodies from the cornea is one of the most painful things one can submit to, yet under the cocaine it

3

is done with such absolute exemption from pain, that the patient scarcely believes the operation ended.

In ulceration of the cornea, and even in iritis, with intense intolerance of light and spasm of the lids, the effect is magical, the spasm and photophobia disappearing in ten or twelve minutes, and not recurring in some instances for several hours. A patient with traumatic iritis, with severe pain and photophobia,—remarked after three or four instillations of the cocaine, that the eye felt easier than for the six weeks previous, and that he "could open his eye for the first time since the injury."

In summing up my clinical experience with the hydrochlorate of cocaine, I have found it most valuable in aiding the examinations of all painful affections of the superficial structures of the eye. In operative procedures, it is exceedingly valuable in cataract operations, removal of foreign bodies from the cornea, strabismus, pterygium, slitting the canaliculi, treating corneal ulcers, and will no doubt become a valuable auxiliary in the enucleation of the ball. In my hands it has not been of much service in operating upon the deeper structures. I would confidently expect great benefit from it in inflammations of the drum membrane (ear ache), and in furuncular inflammations of the external auditory canal, though as yet I have had no opportunity of testing it in these affections. I would suggest that less than a four per cent. solution will not prove satisfactory, and that in some instances, a five per cent. solution will be requisite.

## Figures and Tables

**Figure f1:**